# Case Report: Complete Response of Primary Massive Hepatocellular Carcinoma to Anti-Programmed Death Ligand-1 Antibody Following Progression on Anti-Programmed Death-1 Antibody

**DOI:** 10.3389/fimmu.2021.712351

**Published:** 2021-08-24

**Authors:** Gang Liu, Wenxuan Zhou, Xiaoli Li, Lijie Guo, Tingting He, Juan Zhao, Liansheng Gong

**Affiliations:** ^1^Department of Biliary Surgery, Xiangya Hospital, Central South University, Changsha, China; ^2^Medical Affair Department, OrigiMed, Shanghai, China

**Keywords:** immunotherapy, pembrolizumab, PD-L1, PD-1, HCC, atezolizumab

## Abstract

Hepatocellular carcinoma (HCC) is an aggressive liver tumor that occurs due to chronic liver disease, and it has a high mortality rate and limited treatment options. Immune checkpoint inhibitors have been successfully introduced and used in cancer therapy, among which inhibitors of programmed death ligand-1 (PD-L1) and its receptor programmed death-1 (PD-1) are commonly administered for HCC as combination therapy, including combined anti-angiogenic and immunotherapy combination therapy. We report a case of a primary massive HCC patient with portal hepatic vein tumor thrombus who had a good response to atezolizumab in combination with bevacizumab, following progression of disease on combined immunotherapy with pembrolizumab and lenvatinib. This case demonstrates for the first time that an HCC patient who is resistant to anti-PD-1 antibody immunotherapy can benefit from anti-PD-L1 antibody immunotherapy, providing a potentially promising strategy for the treatment of HCC.

## Introduction

Increasing evidence has shown that immune checkpoint inhibitors are successful as cancer therapy, and these drugs have already become essential for treating a variety of incurable tumors (1). Programmed death-1 (PD-1) and programmed death ligand-1 (PD-L1) are a receptor–ligand system, and the interaction between PD-1 and PD-L1 in tumor microenvironment blocks antitumor immune responses (2). PD-1/PD-L1 pathway blockade is a highly promising therapy and has elicited durable antitumor responses and long-term remission in a subset of patients with a broad spectrum of cancers (3). For example, the use of anti-PD-1 antibodies has improved the survival of hepatocellular carcinoma (HCC) patients (4). Anti-PD-L1 antibodies also provide treatment options for patients with advanced melanoma and HCC (5, 6). Atezolizumab, a PD-L1 antibody, has shown good outcomes among patients with advanced HCC (7). Immunocombination therapy for advanced HCC could shape the direction of first-line treatment (8). Lenvatinib plus pembrolizumab has shown a promising antitumor activity in unresectable HCC (9). Recently, the IMbrave150 trial results demonstrated clinically meaningful benefits of atezolizumab plus bevacizumab in patients’ reported quality of life, function, and disease symptoms as compared with sorafenib, which reinforced the positive benefit-to-risk relationship of combination therapy in patients with unresectable HCC (10). However, there are differences in the efficacy and incidence of immune-related toxicities between PD-1 and PD-L1 inhibitors (11), and therapeutic PD-L1 antibodies are more effective than PD-1 antibodies in blocking PD-1/PD-L1 signaling. Currently, there are limited data on the efficacy and safety of PD-1 inhibitors in the use of PD-L1 inhibitors after disease progression. Herein, we report a case of a primary massive HCC patient who benefited from anti-PD-L1 antibody immunotherapy following progression on anti-PD-1 antibody immunotherapy.

## Case Presentation

**Figure 1:**
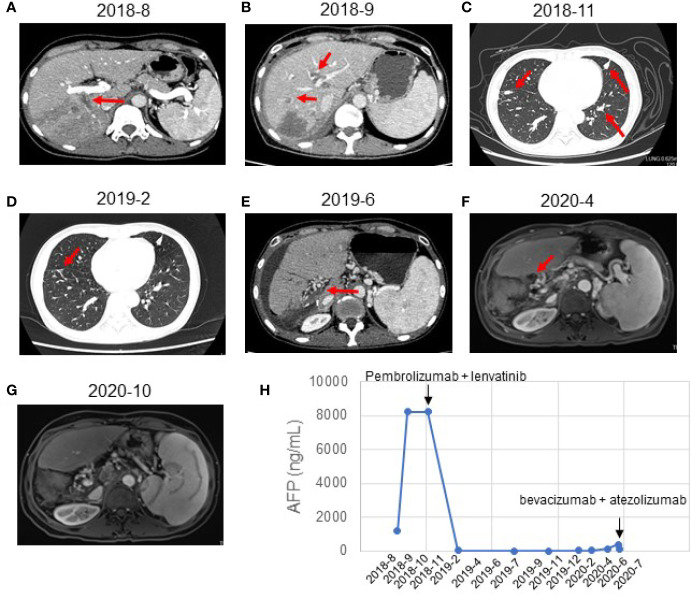
Response evaluation during the clinical course including changes in imaging and quantitative data. **(A)** Preoperative abdominal-enhanced CT revealed a mass in the right posterior lobe of the liver (August 21, 2018). **(B)** CT showed multiple nodules in the liver and tumor thrombus in the right hepatic vein and the left portal vein (September 21, 2018). **(C)** CT indicated new nodules in the lower lobe of the right lung and the upper lobe of the left lung. The multiple original nodules in the lungs had grown (November 2, 2018). **(D)** CT indicated a complete response to pembrolizumab plus lenvatinib (February 2019). The pulmonary nodules had disappeared after treatment. **(E)** CT showed postoperative changes of liver cancer, abnormal density in the liver margin, and a few new inflammatory lesions in both lungs 4 months after treatment with pembrolizumab and lenvatinib (June 2019). **(F)** MRI showed no local active foci and multiple lymph nodes of different sizes in the hilar area and retroperitoneum (April 2020). **(G)** MRI showed a complete response to atezolizumab and bevacizumab (October 2020). The retroperitoneal lymph nodes had disappeared after treatment. **(H)** Graphical depiction of the change in alpha-fetoprotein (AFP) over time.

**Figure 2:**
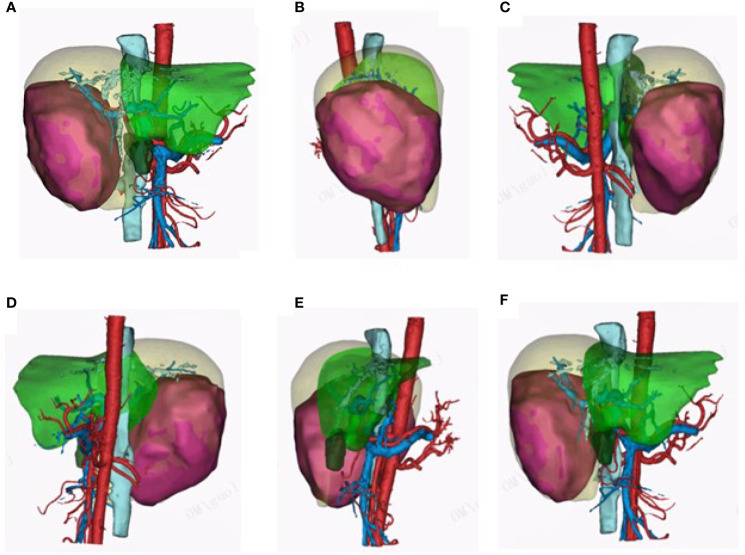
3D visualization pictures. **(A–F)** 3D visualization clearly reconstructed the hepatic vein, hepatic artery, portal vein, and tumor feeding artery. The 3D visualization pictures from different angles are presented.

**Figure 3:**
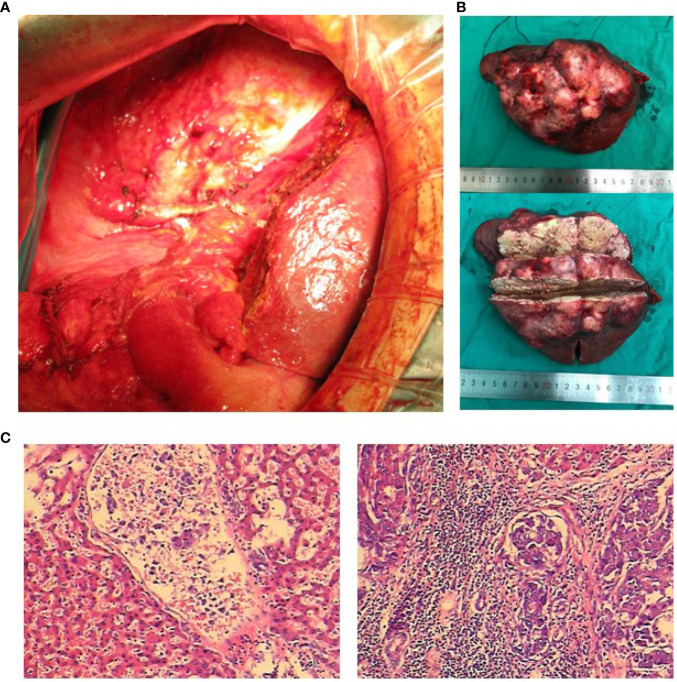
Intraoperative photos, postoperative tumor section, and pathological examination. **(A)** Massive hepatocellular carcinoma (HCC). The liver was examined laparoscopically. **(B)** The resected specimen was a carcinoma with a large amount of necrotic tissue. **(C)** Pathological diagnosis: massive HCC.

A 49-year-old woman presented at a local hospital with right upper abdominal pain, and it was found that the right liver occupies a large position after the examination of abdominal computed tomography (CT) scan and B-ultrasound, and she was therefore referred to our hospital for further examination on August 21, 2018. She had a history of hepatitis B for many years without regular antiviral therapy or other treatments. During hospitalization, she was diagnosed with primary massive HCC with portal hepatic vein tumor thrombus formation, liver cirrhosis, and renal cyst; and her HBV-DNA level was 1.7 × 10^5^ IU/ml. Initial alpha-fetoprotein (AFP) level exceeded the detection limit of 1,200 ng/ml, and CA 125 level was 382.62 U/ml. Abdominal CT revealed a mass within the right hepatic lobe and multiple positive lymph nodes of varying sizes in the hilar area and retroperitoneum. Positron emission tomography–CT revealed a mass shadow with increased glucose metabolism in the lower right lobe of the liver, which was radiologically suspected for HCC, mild cirrhosis, and splenomegaly (**Figure 1A**). We received the results of 3D visualization based on the CT image and performed right hepatic resection to obtain the remnant liver volume on August 29, 2018 (**Figures 2**, **3A, B**). She was diagnosed with primary massive HCC (Barcelona clinic liver cancer (BCLC) stage C, stage IIIA) with portal hepatic vein tumor thrombus. The Eastern Cooperative Oncology Group performance status score was 0, the Child–Pugh score was 8 (stage A), the model for End Stage Liver Disease score was 8, and indocyanine green retention test at 15 min (ICG-15) was 10.5%. Liver functional reserve was assessed preoperatively by both the Child–Pugh classification and ICG-15. The tumor size was 12.1 cm × 11.7 cm, and the volume was 1,085.2 ml/m^2^. The standard liver volume of patient was 1,239.6 cm^3^, and the remnant liver volume was 483 ml/m^2^. The remnant liver-to-standard liver volume ratio was 43.9%, which was sufficient to avoid postoperative liver failure. Histology of the resected tissues indicated poorly differentiated HCC (nodular, 9 × 7.5 × 6 cm) with satellite nodules and necrosis of the tumor and cirrhosis of the liver surrounding the carcinoma (**Figure 3C**). Advanced molecular characterization demonstrated that the tumor was positive for AFP, CK19, HepPar-1, and glypican-3; and negative for Arg-1 and IDH1. The proliferation fraction (Ki67 labeling index) was 70%. The patient was regularly followed up according to the standard protocol of our hospital.

**Figure 4:**
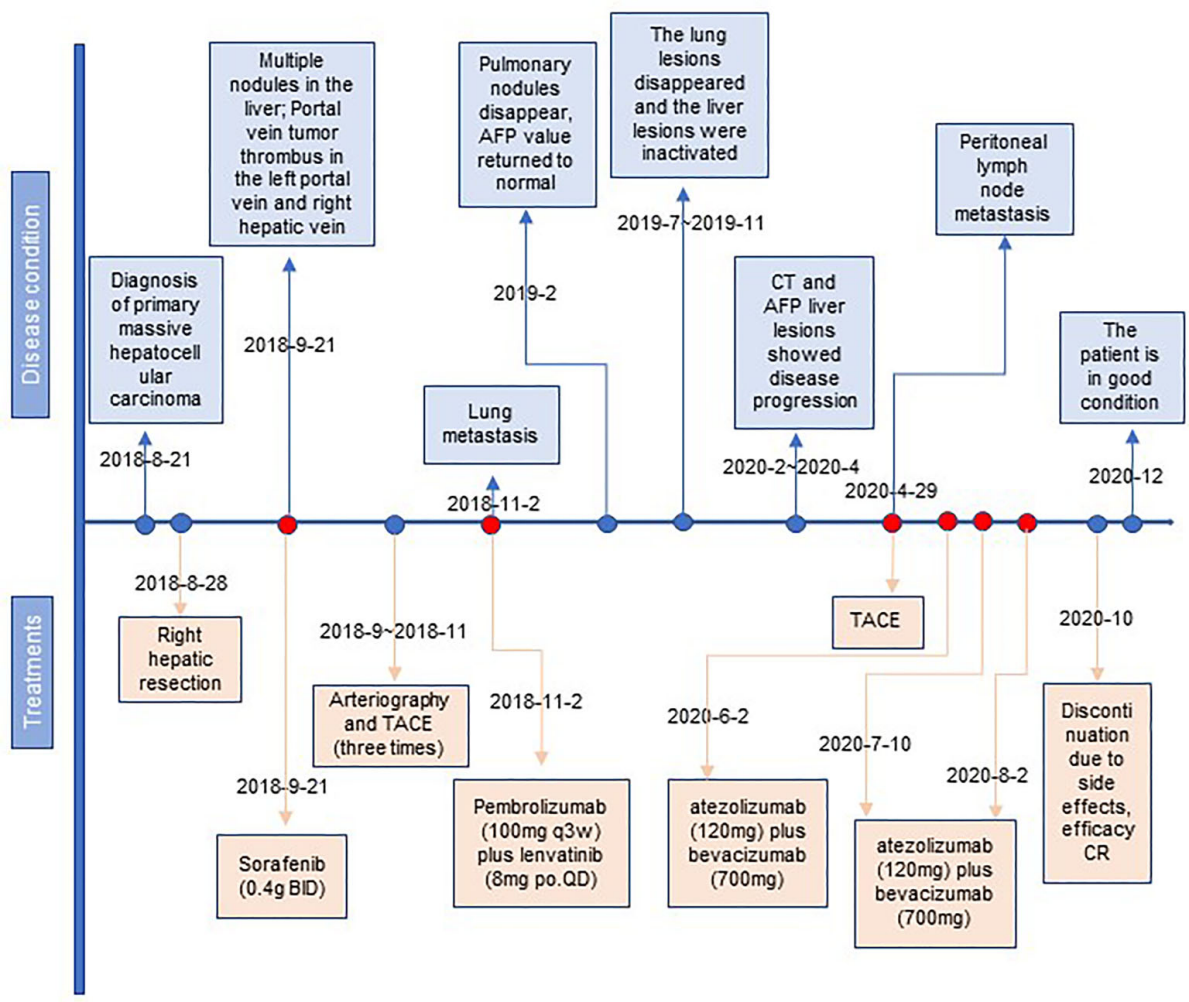
The timeline with therapy and disease status. HCC, hepatocellular carcinoma; TACE, transcatheter arterial chemoembolization; CR, complete response.

A month after the surgery, CT showed multiple nodules in the liver and tumor thrombus in the right hepatic vein and the left portal vein (**Figure 1B**). The AFP reached the highest level (8,221 ng/ml) (**Figure 1H**). We started treatment with sorafenib (0.4 g BID) on September 21, 2018. Transarterial chemoembolization (TACE) was performed three times between September and November 2018. A CT scan on November 2, 2018, revealed disease progression and the development of new pulmonary metastases (**Figure 1C**). She started treatment with pembrolizumab (a PD-1 inhibitor, 100 mg q3w) plus lenvatinib (8 mg po QD) on November 2, 2018. A CT scan in February 2019 showed that the pulmonary nodules had disappeared, and the AFP values had returned to the normal range at the time (**Figures 1D, E**). On July 22, 2019, and November 4, 2019, the patient visited the Beijing Cancer Hospital for examination. Her AFP was within the normal range (2.34 to 3.93), and magnetic resonance imaging showed no active lesions in the local area. In terms of efficacy, the patient achieved a complete response. In February 2020, the patient visited the People’s Hospital of Ningxiang City for examination, and her AFP level had increased to 26.3 ng/ml. Her AFP levels continued to increase (March 11, 2020, 46.63 ng/ml; and April 29, 2020, 134.5 ng/ml), and magnetic resonance imaging showed abdominal lymph node metastases (**Figures 1F, H**). After the patient failed to take TACE treatment on April 29, 2020, we informed the patient and her relatives about the treatment options and alternatives, and she started atezolizumab (a PD-L1 inhibitor, 120 mg) plus bevacizumab (700 mg) on June 2, 2020. After she received atezolizumab plus bevacizumab on July 10 and August 2, 2020, directional radiotherapy was performed. In October 2020, the treatment was discontinued due to side effects, but the patient experienced a complete response based on the 4-month scan (**Figure 1G**). As of December 2020, our follow-up results show that the patient is in good health. Of interest, atezolizumab and bevacizumab combination led to a complete response with disappearance of the retroperitoneal lymph nodes, following progression of disease on pembrolizumab plus lenvatinib, which has shown the hypothesis that switching to a PD-L1 inhibitor from PD-1 inhibitor might overcome potential resistance in the treatment of HCC. At present, the patient is being treated locally with high-intensity focused ultrasound (Hifu) knife, and the follow-up results show that the patient is in good condition. The patient’s course is shown in **Figure 4**.

## Discussion

Surgical intervention for massive HCC through liver lobectomy has historically been the treatment recommendation of choice, particularly as recurrence or local metastasis appears to be rare (12, 13). Our patient was diagnosed with primary massive HCC associated with cirrhosis, which made it extremely difficult to make a surgical choice, as it was likely to cause major hemorrhage and postoperative liver failure (14). Even if surgical intervention is performed, patients with massive HCC may still have a poor prognosis.

The development of immune checkpoint blockade has changed our view of the treatment of a wide variety of malignancies. In 2011, the first immune checkpoint inhibitor (ipilimumab) was approved by the Food and Drug Administration (FDA) for the treatment of melanoma, which started a revolution in immunotherapy for cancer treatment (15). The most common clinically used immunotherapies are PD-1 or PD-L1 inhibitors. PD-1, an important inhibitory receptor, is critical for the maintenance of central and peripheral T-cell tolerance by interacting with its ligands PD-L1 and PD-L2 (2). PD-L1 is universally expressed in normal peripheral tissues, most immune cells, and cancer cells, whereas PD-L2 is mainly expressed on antigen-presenting cells (16). PD-L1 inhibitors can block the engagement of PD-L1 with its receptors PD-1 and CD80 (11). Some investigators found that the PD-L1 antibodies have significantly lower EC50 values than the PD-1 antibodies (11), suggesting that PD-L1 antibodies are perhaps more effective than PD-1 antibodies.

Clinical studies have observed that both nivolumab and pembrolizumab were used as salvage therapy for patients with metastatic HCC who progressed on or after sorafenib treatment (17, 18). As the response rate to PD-1 blockade monotherapy remains low, combination therapy has emerged as a recent trend in cancer treatment (19). Lenvatinib plus pembrolizumab shows promising antitumor activity against HCC. Our patient started pembrolizumab plus lenvatinib and achieved a complete response, but abdominal lymph node metastases occurred 7 months later. Interestingly, atezolizumab and bevacizumab combination led to a complete response, with the disease in the retroperitoneal lymph nodes disappearing, suggesting that switching to a PD-L1 inhibitor from a PD-1 inhibitor might overcome potential resistance in the treatment of HCC.

Currently, there are only a few small retrospective chart reviews and case series in the literature that explore switching from anti-PD-1 antibody therapy to anti-PD-L1 therapy or vice versa (20). In one case report, nivolumab was discontinued in a patient with stable non-small cell lung cancer because of immune-related side effects, and the patient was successfully administered atezolizumab, making the disease stable (21). In another patient with advanced non-small cell lung cancer, atezolizumab therapy led to complete remission following progression on nivolumab in (22). At present, no prospective studies have been conducted to investigate the switch from anti-PD-1 antibody therapy to anti-PD-L1 therapy or vice versa in HCC. In our case, the patient received atezolizumab plus bevacizumab and experienced a complete response. In 2020, Finn et al. examined the combination of atezolizumab and bevacizumab for HCC, and the median progression-free survival (mPFS) for this combination therapy was 6.8 months (23). Patients with advanced HCC who receive ipilimumab with nivolumab or pembrolizumab have a median overall survival (mOS) of 10.9 months (24). A clinical trial enrolled 50 patients treated with anti-PD-1 inhibitor (nivolumab or pembrolizumab) monotherapy, and the mPFS and mOS were 5 and 16.9 months, respectively (25). In addition, treatment with the PD-L1 tyrosine kinase inhibitor durvalumab plus ramucirumab led to mPFS and mOS of 2.6 and 12.4 months, respectively, in HCC patients (26). Our patient received anti-PD-L1 antibody therapy following anti-PD-1 antibody immunotherapy and has sustained a response for 30 months. This is longer than the mOS in these previous studies.

Anti-PD-L1 antibodies may revert resistance to PD-1 inhibition. Our case is a vital addition to the literature, as it suggests that PD-L1 inhibitors may be an effective treatment for patients who have progressed from prior treatment with PD-1 inhibitor. The findings of our case suggest the potential function of switching to anti-PD-L1 treatment in selected patients with primary massive HCC who acquire resistance to PD-1 inhibitors after an initial response. Clinical trials investigating this strategy would be highly beneficial for this increasing cohort of patients. Moreover, to our knowledge, this is the first case to evaluate the outcomes of atezolizumab plus bevacizumab treatment after combined immunotherapy with pembrolizumab and lenvatinib for primary massive HCC, revealing that immunocombination therapy for advanced HCC could shape the direction of first-line or second-line treatment. The limitation is that this is just one individual case; future studies should examine PD-L1 inhibitors in a large number of patients with primary massive HCC that is resistant to PD-1 inhibitors. Over the next few years, it is possible that immunocombination therapy will help more advanced HCC patients and that PD-L1 inhibitor therapy may benefit even more patients who are resistant to PD-1 inhibitors. In conclusion, our case suggests that switching from PD-1 inhibitors to PD-L1 inhibitors may overcome potential resistance to immunotherapy in the treatment of HCC. To the best of our knowledge, this is the first reported case of complete response to anti-PD-L1 immunotherapy following disease progression during anti-PD-1 therapy in HCC.

## Data Availability Statement

The raw data supporting the conclusions of this article will be made available by the authors, without undue reservation.

## Ethics Statement

This study has been approved by Ethic Committee of the Xiangya Hospital. The patient and his relations have signed an informed consent to participate in this study and to publish this report.

## Author Contributions

GL, WZ, XL, and LSG all participated in the management of this case. GL, LJG, TTH, and JZ were in charge of manuscript drafting and data collection. GL and LSG did the modification. All authors contributed to the article and approved the submitted version.

## Funding

This study was funded by the Development of Science and Technology of Hubei Province.

## Conflict of Interest

LJG, TH, and JZ are employees of OrigiMed.

The remaining authors declare that the research was conducted in the absence of any commercial or financial relationships that could be construed as a potential conflict of interest.

## Publisher’s Note

All claims expressed in this article are solely those of the authors and do not necessarily represent those of their affiliated organizations, or those of the publisher, the editors and the reviewers. Any product that may be evaluated in this article, or claim that may be made by its manufacturer, is not guaranteed or endorsed by the publisher.
